# Granuloma Annulare, Autoimmune Thyroiditis, and Lichen Sclerosus in a Woman: Randomness or Significant Association?

**DOI:** 10.1155/2013/289084

**Published:** 2013-05-07

**Authors:** Mariele De Paola, Anastasia Batsikosta, Luca Feci, Mattia Benedetti, Roberta Bilenchi

**Affiliations:** Department of Clinical Medicine and Immunological Sciences, Section of Dermatology, University of Siena, Policlinico “Le Scotte”, 53100 Siena, Italy

## Abstract

We report a 60-year-old Caucasian female with a 2-year history of diffused granuloma annulare (GA), who presented for the simultaneous occurrence of genital lichen sclerosus (LS) and autoimmune thyroiditis (AT). In our opinion this combination is not just coincidental but may share similar immunopathological mechanisms.

## 1. Introduction

GA is one of the “ten noninfectious granulomatous diseases” of the skin, a broad group of distinct reactive inflammatory conditions that share important similarities and that have significant associations with systemic diseases [1]. GA is a benign self-limiting, relatively common dermatosis, typically characterized by an annular arrangement of erythematous or flesh-coloured papules, affecting patients of all ages. Incidence is highest in women, with a ratio of 2.3 to 1.0 over men [2]. The cause of GA is still unknown, but it has been reported following traumas, malignancy, viral infections (including human immunodeficiency virus HIV, Epstein-Barr virus (EBV), and herpes zoster (HZV)), insect bites, and tuberculosis skin tests [3]. The pathogenesis of GA remains still obscure. Possible pathogenetic factors suggested include humoral and delayed type hypersensitivity, vascular damage, metabolic disorder, or primary collagen and/or elastin alteration mediated through an immunologic mechanism [3].

LS is a chronic inflammatory mucocutaneous disease, commonly associated with HLA type B40 related to high incidence of autoimmune diseases, that mainly affects women in the 5th decade but may occur in all age groups, including adolescents and prepubertal children. Its exact prevalence is unknown, but estimates range from 1 : 60 to 1 : 1000. [4]. The etiology is still obscure, although genetic and autoimmune factors, as well as infections, have been implicated in its pathogenesis. It is most commonly seen on the female genital skin, but it also occurs on extragenital areas. Most patients complain of itching and, less frequently, burning sensation, dyspareunia, dysuria, and painful defecation [5].

Autoimmune thyroid disease (AITD) is the most common organ-specific autoimmune disorder, usually resulting in dysfunction (hyperfunction, hypofunction, or both) of the thyroid gland. In some patients, other organ-specific and non-organ-specific autoimmune syndromes are associated with autoimmune thyroid disease, including pernicious anemia, vitiligo, myasthenia gravis, primary adrenal autoimmune disease, celiac disease, rheumatoid arthritis, or lupus [6]. We report a case of GA, AT, and LS in a woman and discuss their comorbidity.

## 2. Case Presentation

**Figure 1:**
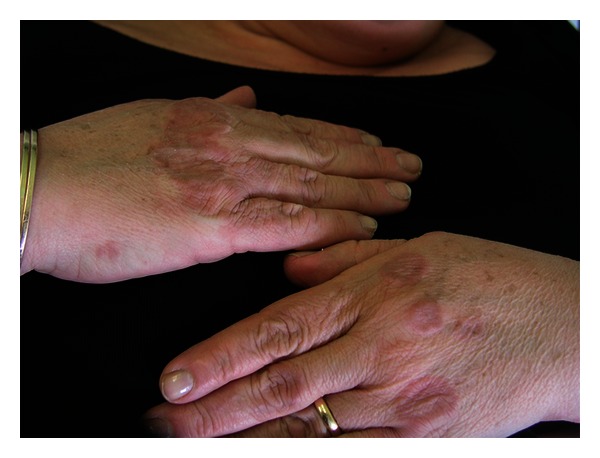
Woman with annular arrangement of erythematous and flesh-coloured papules at her hands.

**Figure 2:**
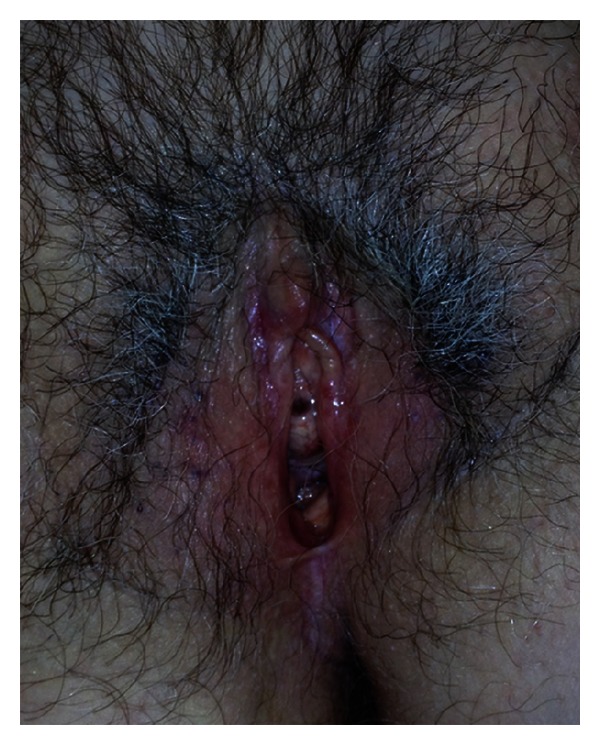
Lichen sclerosus with porcelain-white atrophic scarring, erosions, and hemorrhages in the genital area.

**Figure 3:**
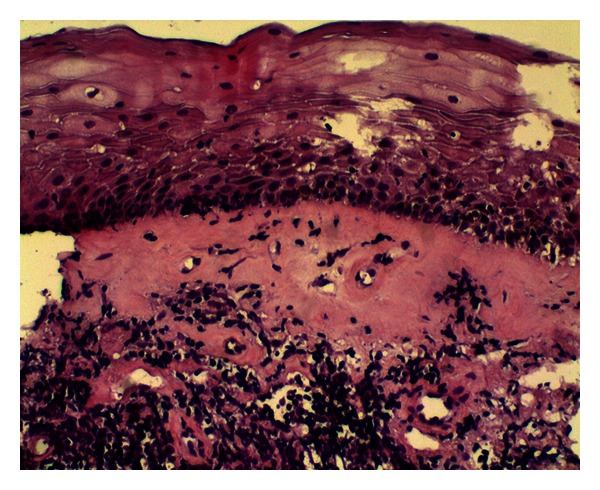
Epidermal thinning, dermic broad bundles of hyalinized collagen, and band-like lymphocytic infiltrate (haematoxylin eosin).

**Figure 4:**
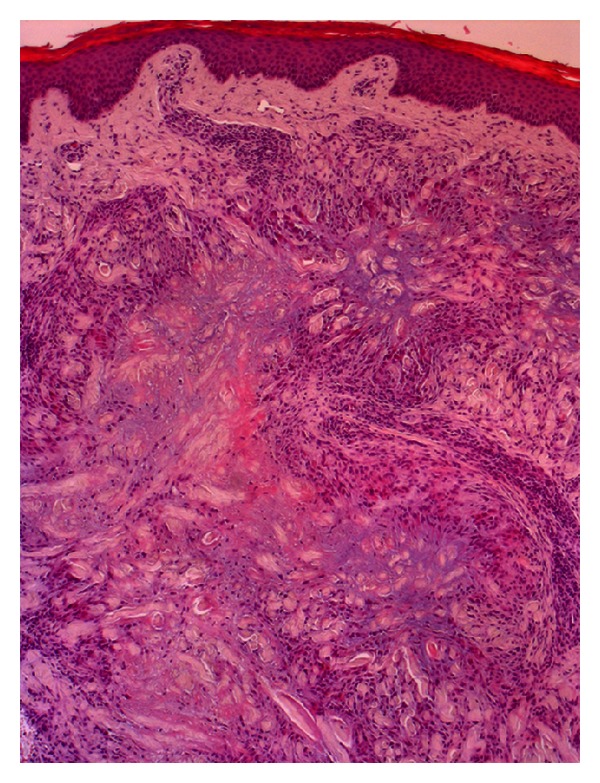
Normal epidermis. Palisaded granulomas of dermis with epithelioid histiocytes around a central zone of mucin (haematoxylin eosin).

A 60-year-old Caucasian female presented with a history of 2-year annular arrangement of erythematous papules at the right arm and hands (Figure 1), histologically confirmed as GA (Figure 2). The patient was treated topically with clobetasol propionate ointment and pimecrolimus cream for three months with partial resolution. Two years after, the patient developed vulvar LS (Figures 3 and 4). On admission, routine hematologic and chemistry analyses were in the normal range with the exception of positive speckled pattern (1 : 160 titer) antinuclear antibody (ANA). Detection of serum antibodies to *B. burgdorferi* was negative. Thyroid function tests showed subclinical autoimmune hypothyroidism with elevated thyroid-stimulating hormone (TSH) values (7.7 UL/mL showed subclinical autoimmune hypothyroidism 421 IU/ml; antimicrosomal of 43 IU/mL). Thyroid ultrasound revealed multiple thyroid nodules. The patient was managed with methylprednisolone 4 mg per day. After 2 months there was a favorable improvement of skin symptoms with incomplete clearing of lesions.

## 3. Discussion 

GA associated with LS and autoimmune thyroiditis have been never reported to occur simultaneously while evidence of improvement to treatment with systemic steroids of a single disease is often reported. Some studies have indicated an association of skin granulomatous disease like granuloma annulare and autoimmune diseases like diabetes mellitus or Sjögren's syndrome or thyroid diseases. Some other studies have reported cooccurrence of LS with autoimmune diseases including vitiligo, Hashimoto's thyroiditis and type 1 diabetes [7]. In the literature, it has been raised that this association is more than coincidence, and it suggests an autoimmune basis for these conditions (Table 1).

Granuloma annulare is a benign disease of unknown etiology with a lymphocyte-mediated hypersensitivity type IV mechanism where an immunologic cell-mediated process or a primary collagen and/or elastin destruction have often been suggested [8]. Lichen sclerosus is a chronic lymphocyte-mediated inflammatory skin disease, for which increasing evidence suggests an underlying autoimmune mechanism mainly in female genetically predisposed patients with HLA-DQ7 antigen; in fact antibodies against the basement membrane zone, chiefly Binding Protein (BP)180 and BP 230, have been found in 30% of sera of patients with LS, and a high proportion of patients (up to 80%) have specific antibodies targeting extracellular matrix protein-1 [9]. On these basis we think, according to the literature, that the origin is most likely immunologic associated with a mechanism of inheritance. Interestingly, there is also evidence linking GA and autoimmune thyroiditis/insulin-dependent diabetes mellitus (IDDM), and evidence linking LS and AT that come from both limited epidemiologic data and clinical observations.

Actually, there is no definitive hypothesis that could explain the coexistence of GA, LS, and AT. However, we believe that in our patient, GA, with lymphocyte-mediated hypersensitivity type IV mechanism and a cell-mediated immune process, could contribute to trigger an organ-specific auto-immune response also in the genital mucosa or gland tissue, leading to the development of LP lesions and autoimmune thyroiditis. Further study is necessary to determine whether the LS, AT, and GA in our patient were an isolated coincidence or there is a causative link between those. We speculate that the pathogenesis of GA might play an important role in the genesis of the other diseases in this case.

Our case suggests that AT, GA, and LS may be closely related and that GA and LS should be recognized as two cutaneous manifestations associated with autoimmune diseases. However, we cannot exclude that comorbidity is exclusively a coincidence and further investigations are needed to better understand etiopathogenesis and develop more specific therapy.

## Figures and Tables

**Table 1:** Granulomatous diseases and autoimmune diseases associations.

Author/year	Type of study	Associated disease/s
Studer et al./1996 [10]	Retrospective study	Diabetes mellitus 10/84 cases
Erkek et al./2001 [11]	Case report	Insulin-dependent diabetes mellitus
Vázquez-López et al./2003 [12]	Case-control study	Autoimmune thyroditis 3/24
Tallab/2004 [13]	Case report	Autoimmune thyroditis
Goucha et al./2008 [14]	Retrospective study (GA)	Diabetes mellitus 9/35 cases Thyroiditis 2/35 cases
Davison et al./2010 [15]	Pediatric case report	2/2 cases of juvenile insulin-dependent diabete mellitus
Sumikawa et al./2010 [16]	Case report	1 case of Sjögren's syndrome
Sehgal et al./2011 [17]	Review of clinical case histories (GA)	100 patients with generalized granuloma annulare; 13 patients were found to have thyroid disease, while diabetes was diagnosed in 21 patients.

## References

[1] Hawryluk EB, Izikson L, English JC (2010). Non-infectious granulomatous diseases of the skin and their associated systemic diseases: an evidence-based update to important clinical questions. *The American Journal of Clinical Dermatology*.

[2] Dahl MV, Fitzpatrick TB, Eisen AZ, Wolff K, Freedberg IM, Austin KF (2003). Dahl MVGranuloma annulare. *Dermatology in General Medicine*.

[3] Stewart LR, George S, Hamacher KL, Hsu S (2011). Granuloma annulare of the palms. *Dermatology Online Journal*.

[4] Murphy R (2010). Lichen sclerosus. *Dermatologic Clinics*.

[5] Fistarol SK, Itin PH (2013). Diagnosis and treatment of lichen sclerosus : an update. *The American Journal of Clinical Dermatology*.

[6] Trbojević B, Djurica S (2005). Diagnosis of autoimmune thyroid disease. *Srpski arhiv za celokupno lekarstvo*.

[7] Meffert JJ, Davis BM, Grimwood RE (1995). Lichen sclerosus. *Journal of the American Academy of Dermatology*.

[8] Sniezek PJ, Debloom JR, Arpey CJ (2005). Treatment of granuloma annulare with the 585 nm pulsed dye. *Dermatologic Surgery*.

[9] Howard A, Dean D, Cooper S, Kirtshig G, Wojnarowska F (2004). Circulating basement membrane zone antibodies are found in lichen sclerosus of the vulva. *The Australasian Journal of Dermatology*.

[10] Studer EM, Calza AM, Saurat JH (1996). Precipitating factors and associated diseases in 84 patients with granuloma annulare: a retrospective study. *Dermatology*.

[11] Erkek E, Karaduman A, Bükülmez G, Sentürk N, Ozkaya O (2001). An unusual form of generalized granuloma annulare in a patient with insulin-dependent diabetes mellitus. *Acta Dermato-Venereologica*.

[12] Vazquez-Lopez F, Pereiro M, Haces JAM (2003). Localized granuloma annulare and autoimmune thyroiditis in adult women: a case-control study. *Journal of the American Academy of Dermatology*.

[13] Tallab TM (2004). Localized granuloma annulare and autoimmune thyroiditis in a Saudi patient: report of a new case. *West African Journal of Medicine *.

[14] Goucha S, Khaled A, Kharfi M (2008). Granuloma annulare. *G Giornale Italiano di Dermatologia e venereologia : organo ufficiale, Società Italiana di Dermatologia e Sifilografia*.

[15] Davison JE, Davies A, Moss C, Kirk JM, Taibjee SM, Agwu JC (2010). Links between granuloma annulare, necrobiosis lipoidica diabeticorum and childhood diabetes: a matter of time?. *Pediatric Dermatology*.

[16] Sumikawa Y, Ansai S, Kimura T, Nakamura J, Inui S, Katayama I (2010). Interstitial type granuloma annulare associated with Sjögren's syndrome. *The Journal of Dermatology*.

[17] Sehgal VN, Bhattacharya SN, Verma P (2011). Juvenile, insulin-dependent diabetes mellitus, type 1-related dermatoses. *Journal of the European Academy of Dermatology and Venereology*.

